# Myeloid Dendritic Cells Are Enriched in Lymph Node Tissue of Early Rheumatoid Arthritis Patients but not in At Risk Individuals

**DOI:** 10.3390/cells8070756

**Published:** 2019-07-20

**Authors:** T.H. Ramwadhdoebe, M.I. Ramos, K.I. Maijer, K.P. van Lienden, M. Maas, D.M. Gerlag, P.P. Tak, M.C. Lebre, L.G.M. van Baarsen

**Affiliations:** 1 Department of Rheumatology & Clinical Immunology, Amsterdam UMC, University of Amsterdam, Meibergdreef 9, 1105 AZ Amsterdam, The Netherlands; 2 Department of Experimental Immunology, Amsterdam Infection & Immunity Institute, Amsterdam UMC, University of Amsterdam, Meibergdreef 9, 1105 AZ Amsterdam, The Netherlands; 3 Department of Radiology, Amsterdam UMC, University of Amsterdam, Meibergdreef 9, 1105 AZ Amsterdam, The Netherlands

**Keywords:** dendritic cells, lymphoid tissue, at-risk individuals, rheumatoid arthritis

## Abstract

Lymph nodes (LNs) are highly organized structures where specific immune responses are initiated by dendritic cells (DCs). We investigated the frequency and distribution of human myeloid (mDCs) and plasmacytoid (pDCs) in LNs and blood during the earliest phases of rheumatoid arthritis (RA). We included 22 RA-risk individuals positive for IgM rheumatoid factor and/or anti-citrullinated protein antibodies, 16 biological-naïve RA patients and 8 healthy controls (HCs). DC subsets (CD1c^+^ mDCs and CD304^+^ pDCs) in LN tissue and paired peripheral blood were analyzed using flow cytometry and confocal microscopy. In blood of RA patients a significant decreased frequency of pDCs was found, with a similar trend for mDCs. In contrast, mDC frequencies were higher in RA compared with HCs and RA-risk individuals, especially in LN. Frequency of mDCs seemed higher in LNs compared to paired blood samples in all donors, while pDCs were higher in LNs only in RA patients. As expected, both mDCs and pDCs localized mainly in T-cell areas of LN tissue. In conclusion, compared with RA-risk individuals, mDCs and pDCs were enriched in the LN tissue of early-RA patients, while their frequency in RA-risk individuals was comparable to HCs. This may suggest that other antigen-presenting cells are responsible for initial breaks of tolerance, while mDCs and pDCs are involved in sustaining inflammation.

## 1. Introduction

Dendritic cells (DCs) are professional antigen-presenting cells that specialize in the uptake of antigens and their transport from peripheral tissues to lymphoid organs. Because of their capacity to stimulate naive T cells, DCs have a central role in the initiation of immune responses and are considered promising tools and targets for immunotherapy [1,2]. Emerging data suggest a role for DCs in initiating and perpetuating autoimmune diseases [3,4]. In particular, in rheumatoid arthritis (RA) it has been proposed that DCs present arthritogenic antigens to T cells [5]. In addition, two main DC subsets, myeloid or conventional (mDCs) and plasmacytoid (pDCs), with distinct functions have been the focus of much attention [6]. In this respect, it was shown that mDCs and pDCs are decreased in RAperipheral blood [7], possibly due to their accumulation at the site of inflammation (the synovium) [8,9]. However, it is unclear whether in RA these DC subsets also accumulate in the lymph nodes (LNs) where they may present (auto)antigens to T cells, and whether this accumulation can be found already in RA-risk individuals positive for autoantibodies.

A dual role has been described for DCs in autoimmunity [10]. In lymphoid tissues, DCs can contribute to peripheral tolerance by promoting regulatory T cell differentiation or promoting T cell unresponsiveness [11,12]. On the other hand, DCs can promote or induce autoimmunity through increased migration, self-antigen presentation or cytokine release [4,13] as a response to an imbalance of pro- or anti-inflammatory cytokines.

This dual role of DCs in autoimmunity has been studied in several experimental arthritis models [14,15,16,17]. pDCs have been described as contributing to self-tolerance in an ovalbumin (OVA)-induced arthritis model where depletion of pDCs before the onset of disease using a specific antibody caused enhanced severity of articular pathology and increased autoimmune responses against type II collagen [16]. This suggests a regulatory role for pDCs in preventing autoimmunity. On the other hand, mDCs might contribute to experimental arthritis induction after priming with autoantigens [18].

In RA patients, mDCs derived from synovial fluid are capable of producing pro-inflammatory cytokines that promote T cell responses [8,19]. In contrast, pDCs in peripheral blood of RA patients display a tolerogenic phenotype and can potentially suppress the proliferation of autoreactive T cells in vitro [20]. However, synovial tissue-derived pDCs may locally produce type I interferon and thereby promote autoantibody production by B cells [21]. In addition, synovial tissue mDCs and pDCs display an immature phenotype indicating that they might be recently activated or that mature DCs have migrated from the synovial tissue towards lymphoid tissue (reviewed in [9]).

To study the possible contribution of different DC subsets during the earliest phases of RA, we analyzed the frequencies of both mDCs and pDCs in paired peripheral blood and LN tissue (inguinal LNs) samples from healthy controls (HCs), individuals at risk for developing arthritis by having systemic autoimmunity (RA-risk individuals) and early-RA patients. Moreover, we investigated the location of mDCs and pDCs within LN tissue to further delineate the possible interactions with T and B cells.

## 2. Methods

### 2.1. Study Subjects and Lymph Node Biopsy Sampling

We included 22 individuals with arthralgia and elevated IgM-rheumatoid factor (RF) and/or anti-cyclic citrullinated peptide antibody (ACPA) serum levels, without any evidence of arthritis upon clinical examination (RA-risk individuals, phase c/d) [12]. IgM-RF was measured using IgM-RF ELISA (Hycor Biomedical, Indianapolis, IN, USA (ULN 49 IU/mL)). ACPA was measured using anti-CCP2 ELISA CCPlus (Eurodiagnostica, Nijmegen, the Netherlands (ULN 25 kAU/L)). After a median follow-up time of 25.6 months (13.6–38.7 interquartile range (IQR)) none of these RA-risk individuals had developed RA yet despite the presence of autoantibodies. However, we expect that approximately 26% of these individuals will develop arthritis within five years [22]. These individuals are termed RA-risk individuals, as recommended by the Study Group for Risk Factors for RA (SGRFRA) under the auspices of the EULAR (the European League Against Rheumatism) Standing Committee of Investigative Research (ESCIR) [23]. Furthermore, we included 16 RA patients with established disease based on fulfillment of the American College of Rheumatology and European League Against Rheumatism (ACR/EULAR) 2010 criteria [24], as well as eight healthy controls without any joint complaints and without elevated IgM-RF and/or ACPA levels. These healthy controls did not have a recent history of viral infection, possessed no autoimmunity or malignancy and had no present or previous use of DMARDs (disease-modifying anti-rheumatic drugs), biologicals or experimental drugs. The study was performed according to the principles of the Declaration of Helsinki, approved by the institutional medical ethical review board of the Academic Medical Centre, and all study subjects gave written informed consent. All study subjects underwent an ultrasound-guided inguinal LN needle core biopsy as previously described [25]. Several lymph node biopsies were put through a 70-μm cell strainer (BD Falcon, San Jose, CA, USA) to obtain a single cell suspension, which was immediately analyzed by flow cytometry. On the day of LN sampling, none of the donors showed signs of an infection. Table 1 shows the demographics of the included subjects.

### 2.2. Isolation of Peripheral Blood Mononuclear Cells and Flow Cytometry Analysis

Paired peripheral blood mononuclear cells (PBMC) were isolated using standard density gradient centrifugation with lymphoprep (Nycomed AS, Oslo, Norway) and stored in liquid nitrogen until further use. After thawing, cells were stained extracellularly for 30 min at 4 °C in PBS containing 0.01% NaN_3_ and 0.5% BSA with directly labeled antibodies against: HLA-DR APC-H7, CD45 V500 (all from BD Biosciences, San Jose, CA, USA); CD1c/BDCA1-Fitc, CD304/BDCA4-APC, CD304-PE (all from Miltenyi Biotec, Leiden, the Netherlands); CD304 Percp Cy5.5 (Biolegend, Uithoorn, the Netherlands); and lineage-alexa 700 (AbD Serotec, Oxford, UK). In PBMC, Lineage^-^HLA-DR^+^ CD1c^+^ or CD304^+^ were considered as mDCs or pDCs, respectively. In LNs, CD45^+^HLA-DR^+^ CD1c^+^ or CD304^+^ were considered as mDCs or pDCs, respectively [26,27]. Cells were acquired on a FACS Canto II (BD Biosciences) and data were analyzed using FlowJo software (FlowJo, Ashland, OR, USA). Data were plotted as frequency of positive cells.

### 2.3. Immunofluorescence Microscopy

Freshly collected LN biopsies were embedded in OCT tissue TEK and stored in liquid nitrogen. Frozen sections were cut (5 μm) using a cryostat. Sections were stored at −80 °C until further use. For staining, sections were thawed and air dried at room temperature and subsequently fixed with acetone. Sections were washed and stained with primary antibodies diluted in PBS/1%, BSA/10% and normal human serum (NHS; Lonza, Basel, Switzerland) overnight at 4 °C: CD1c/BDCA1-Fitc (mouse anti-human IgG2a; Miltenyi Biotec) or CD303/BDCA2 (mouse anti-human IgG1; Miltenyi Biotec), CD19-biotin (mouse anti-human IgG1; Biolegend) and CD3 (rabbit anti-human; Thermo Scientific, Waltham, MA, USA). Isotype controls were as follows: mouse IgG2a-Fitc (Biolegend), mouse IgG1-biotin (Biolegend), rabbit IgG (Dako Cytomation, Heverlee, Belgium) and mouse IgG1 (Dako Cytomation). After washing with PBS, (directly labeled) secondary antibodies were incubated for 30 min in PBS/1%, BSA/10% and NHS: goat anti-mouse IgG2a, Steptavidin alexa fluor 633, goat anti-rabbit alexa fluor 546 and goat anti-mouse IgG1 alexa 488. The combination of CD303/BDCA2, CD3 and CD19 was stained using a five-step protocol including an extra blocking step with normal mouse serum (Sanquin, Amsterdam, The Netherlands). The combination of CD1c/BDCA1-Fitc, CD3 and CD19 was stained using a two-step protocol. After washing with PBS, slides were covered with Vectashield containing DAPI (Vector Laboratories, Burlingame, CA, USA) and analyzed on a Confocal imaging microscope (Leica Microsystems, Wetzlar, Germany).

### 2.4. Statistics

Not-normally distributed data were presented as median with interquartile range (IQR) and analyzed using a Kruskal–Wallis test followed by a post Dunn’s multiple comparisons test. Paired data were analyzed with a Wilcoxon matched pairs test. Correlations were calculated using Spearman’s rho. All statistical analyses were performed using GraphPad Prism Software (version 8, GraphPad Software, Inc. La Jolla, CA, USA). *p*-values ≤ 0.05 were considered statistically significant.

## 3. Results

### 3.1. CD1c^+^ mDCs are Enriched in Human LN Tissue of Early-RA Patients

**Figure 1:**
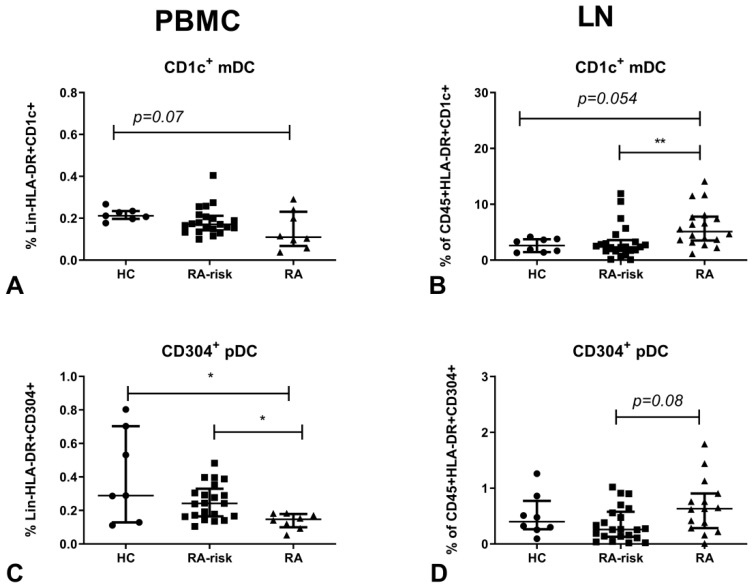
Myeloid (mDC and plasmacytoid(p)DC frequencies in lymphoid tissue and peripheral blood. Frequencies of mDCs (**A**,**B**) and pDCs (**C**,**D**) are determined in peripheral blood mononuclear cells (PBMC; A,C) and lymphoid tissue (LN; B,D). For PBMC, the frequencies are plotted as frequencies of the lineage (Lin)^-^HLA-DR^+^ population. For lymph nodes (LNs) the frequencies are plotted as frequencies of the CD45^+^HLA-DR+ population. PBMC: HC (*n* = 7), RA-risk (*n* = 21) and RA (*n* = 8). LN: HC (*n* = 8), RA-risk (*n* = 22), RA (*n* = 15 or 18). Data are presented as median with interquartile range (IQR). For statistical analysis, a Kruskall–Wallis test was performed and significant differences were determined using a post Dunn’s multiple comparisons test and indicated as * *p* < 0.05 or ** *p* < 0.01.

We determined the frequencies of CD1c^+^ mDCs and CD304^+^ pDCs in both the blood and LN tissue of healthy controls (HCs), RA-risk individuals and early-RA patients by flow cytometry. As expected [7], the frequency of CD1c^+^ mDCs (Figure 1A) in PBMC of early-RA patients was decreased compared with the HCs. In lymphoid tissue, the frequency of CD1c^+^ mDCs was significantly increased in early-RA patients compared with RA-risk individuals, and a clear trend towards increased frequency in early-RA patients compared with the HCs was observed (Figure 1B). The frequency of CD304^+^ pDCs in PBMC was significantly decreased in early-RA patients compared with RA-risk individuals and HCs (Figure 1C), while in lymphoid tissue a trend towards increased frequency of CD304^+^ pDCs was observed in early-RA patients compared with RA-risk individuals (Figure 1D). In blood and lymph node tissue of RA-risk individuals, frequencies of mDCs and pDCs were comparable to healthy controls.

### 3.2. Compared to Blood, CD304^+^ DC Frequencies are Higher in Lymphoid Tissue of Early-RA Patients

**Figure 2:**
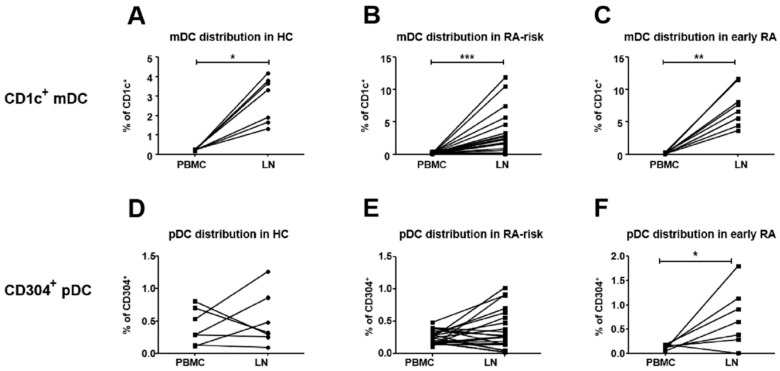
Frequency of mDCs and pDCs in human lymphoid tissue versus peripheral blood. Paired analysis was performed to determine the distribution of mDCs (upper panel, **A**, **B** and **C**) and pDCs (lower panel, **D**, **E** and **F**) in PBMC and LNs of HCs (**A** and **D**), RA-risk (**B** and **E**) and RA (**C** and **F**) individuals. HC (*n* = 7), RA-risk (*n* = 21) and RA (*n* = 8). DCs in PBMC were defined as Lineage^-^HLA-DR^+^CD1c^+^ or CD304^+^, while DCs in LN tissue were defined as CD45^+^HLA-DR^+^CD1c^+^ or CD304^+^. For statistical analysis, paired data were analyzed with a Wilcoxon matched pairs test (* *p* < 0.05 or ** *p* < 0.01, *** *p < 0.001*).

Next we investigated how the relative percentage of CD1c^+^ mDCs and CD304^+^ pDCs within the total lymphoid tissue DCs related to their relative percentage found in blood circulating DCs. Compared to blood, CD1^+^ mDCs accumulated in lymphoid tissue in all three study groups (Figure 2A–C). In contrast, frequencies of CD304^+^ pDCs were similar in lymphoid tissue and blood of the HCs (Figure 2D) and RA-risk individuals (Figure 2E), while in early-RA patients CD304^+^ pDCs accumulated in lymph node tissue compared to blood (Figure 2F).

### 3.3. Both mDCs and pDCs Localize Mainly in T Cell Areas of Human Lymphoid Tissue

**Figure 3:**
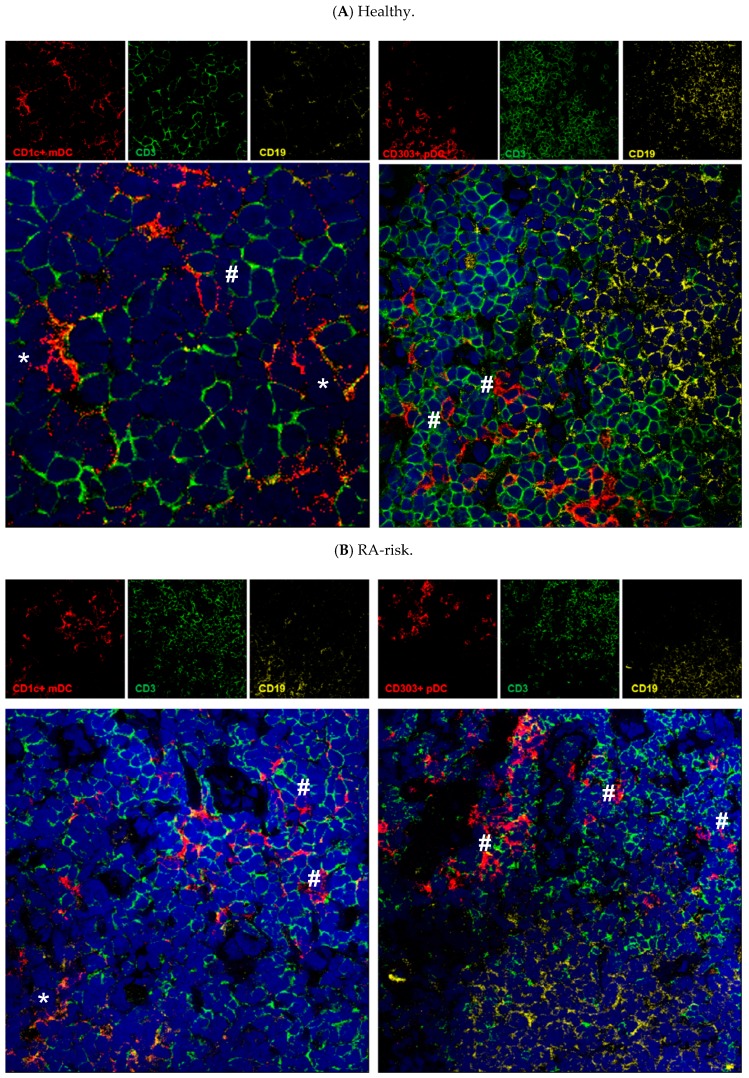

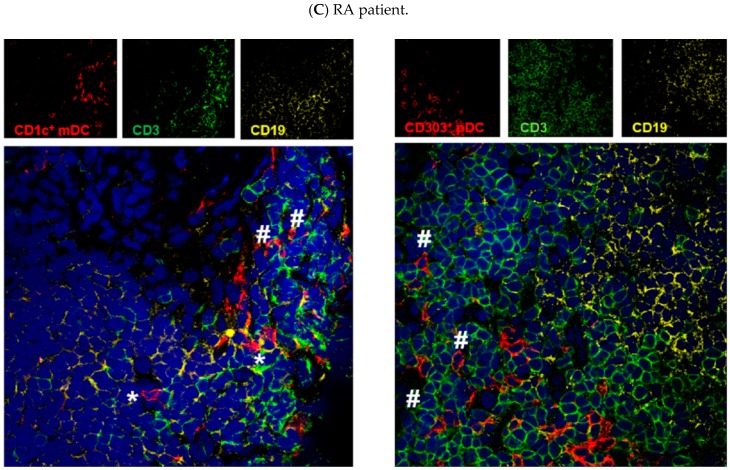
Localization of mDCs and pDCs in human lymphoid tissue. Lymph node tissue sections from a healthy control (**A**), RA-risk individual (**B**) and RA patient (**C**) stained for mDCs (left panels) and pDCs (right panels) using BDCA1 or BDCA2 antibodies (indicated in red). CD19+ B cell areas are indicated in yellow and CD3^+^ T cell areas are indicated in green. In 63x zoom images # represent DCs in close proximity to T cells. * represents DCs in close proximity to B cells.

Next we investigated the location of CD1c^+^ mDCs and CD303^+^/BDCA2^+^ pDCs in lymphoid tissue. CD1c^+^ mDCs are located mainly in the T cell areas and in T cell areas close to B cell follicles (Figure 3, indicated with #). As expected, a few CD1c^+^ mDCs were found within B cell areas (indicated with *). CD304^+^ pDCs were mainly localized in T cell areas of lymph nodes (Figure 3, indicated with #). In LN tissue of healthy controls (Figure 3A), RA-risk individuals (Figure 3B) and RA patients (Figure 3C), mDCs and pDCs were found at similar locations.

## 4. Discussion

In this study, we analyzed for the first time the frequencies of CD1c^+^ mDCs and CD304^+^ pDCs in paired blood and lymphoid tissue samples obtained from healthy controls, individuals at risk for RA and early-RA patients. We observed a trend towards decreased frequency of mDCs in the blood of early-RA patients compared with the HCs, and a significant decreased frequency of pDCs in the blood of early-RA patients compared with the HCs and RA-risk individuals. The frequency of mDCs and pDCs was higher in the LN tissue of early-RA patients compared with RA-risk individuals. In contrast, the frequencies of mDCs and pDCs in RA-risk individuals for LNs and blood were on average similar to frequencies observed in healthy controls. Compared to blood, mDCs were more frequent in LN tissue of the HCs, RA-risk and early-RA patients, while lymph node pDCs were increased only in early-RA patients. As expected [28], both mDCs and pDCs localize mainly in T cell areas of LN tissue.

When activated, DCs home into the T cell zones of lymphoid organs where they accumulate and interact with T cells in order to initiate specific immune responses. The observed increased frequencies of both mDCs and pDCs in arthritic lymphoid tissue may reflect presentation of (arthritogenic) antigens to (autoreactive) T cells. Besides the presentation of (auto)antigens, DCs might contribute to inflammation and B cell differentiation in lymphoid tissue through other mechanisms. pDCs have been described to enter lymph nodes through high endothelial venules and accumulate in inflamed lymph nodes where they can produce large amounts of type I interferon upon T cell activation [29]. In addition, type I interferon signaling in lymph nodes stimulates the development of lymph node follicular T helper cells [30], and could therefore indirectly contribute to increased B cell responses in peripheral lymphoid organs. Importantly, pDCs have been shown to drive B cell differentiation into plasma cells in a T cell-dependent [31] and a T cell-independent way [32] (through TLR9 triggering and CpG-BCR ligation). Our data show that pDCs in lymphoid tissue of RA patients mainly localize near T cells, suggesting that mainly T cell-dependent mechanisms of action play a role during the established phase of disease. In contrast to mDCs, which accumulated in the lymphoid tissue of HCs, RA-risk and early-RA patients, accumulation of pDCs in lymphoid tissue was only observed in patients with established RA. This observation may be related to accumulation of this DC subset upon inflammation [29]. mDCs enter lymph nodes through afferent lymphatics [33], and have been described as producing large amounts of IL-12 [34], which directly affects B cells and stimulates plasma cell differentiation or can induce the differentiation of follicular T helper cells in lymphoid tissue [35]. In general, lymphoid tissue in healthy individuals, RA-risk individuals and early-RA patients contains more mDCs than pDCs (Figure 1). This suggests that mDCs may play an important role in the initiation of the disease by presenting (auto)antigens to CD8 T cells [36,37,38]. Related to this, our data show that in early RA, mDCs localized in T cell areas and in close proximity to B cells, suggesting that mDCs may form a complex with B and T cells and activate both [39]. Together with the increased frequencies of both mDCs and pDCs in early-RA patients, a general increase in peripheral lymphoid tissue immune responses is plausible.

Of importance, our data show that the frequency of mDCs and pDCs in blood and lymph nodes is not changed during the RA-risk phase of disease, despite the presence of autoantibodies. Assuming that the autoantibodies are produced upon antigen presentation, this may suggest that either the function of mDCs and pDCs is impaired, or that maybe other antigen-presenting cells are responsible for the initial break of tolerance, while mDCs and pDCs are important players in the inflammatory phase of disease (in which increased frequencies have been observed). Additional studies are needed to elucidate the functional role of mDCs and pDCs during RA development. It will be of interest to study possible additional antigen-presenting cell candidates like B cells and follicular DC, but also stromal cells in lymphoid tissue during the earliest phases of RA. Moreover, longer follow up of these RA-risk individuals will be required to show which changes in lymph node tissue immunopathology are related to development of disease.

## Figures and Tables

**Table 1:** Baseline characteristics of healthy controls (HCs), rheumatoid arthritis (RA)-risk individuals and early-RA patients. Categorical variables: n (%). Continuous variables (data not normally distributed): median interquartile range (IQR). ACPA, anticitrullinated protein antibodies; ESR, erythrocyte sedimentation rate; CRP, C-reactive protein; IgM-RF, IgM rheumatoid factor; 68 TJC, tender joint count of 68 joints; 66 SJC, swollen joint count of 66 joints; DAS, disease activity score; nd, not done.

Demographics	HCs	RA-Risk	Early Arthritis (RA)
	*n* = 8	*n* = 22	*n* = 16
Sex, female (%)	6 (75)	18 (82)	10 (63)
Age (years) (median (IQR))	34.0 (28.0–41.8)	49.0 (43.5–57.5)	49.0 (38.0–57.0)
IgM-RF positive (n (%))	0 (0)	9 (41)	15 (94)
IgM-RF level (kU/L) (median ((IQR))	1.0 (1.0–1.5)	21.0 (3.0–117.5)	182.0 (45.5–312.0)
ACPA positive (n (%))	0 (0)	13 (59)	14 (88)
ACPA level (kAU/L) (median (IQR))	2.5 (1.8–3.3)	47.0 (4.5–202.0)	119.0 (22.5–865.5)
IgM-RF and ACPA both pos. (n (%))	0 (0)	0 (0)	13 (81)
ESR (mm/h) median (IQR))	nd	8.0 (3.5–11.0)	12.0 (6.5–22.0)
CRP (mg/L) (median (IQR))	0.7 (0.4–1.1)	1.9 (0.9–4.3)	4.6 (1.9–9.1)
68 TJC (n) (median (IQR))	0 (0)	2.0 (1.0–3.0)	14.0 (5.0–23.5)
66 SJC (n) (median (IQR))	0 (0)	0 (0)	7.0 (4.5–11.0)
DAS 28 (median (IQR))			4.6 (3.6–5.8)
